# Planning and effectiveness of intensive rehabilitation as a treatment for a patient with neurosarcoidosis: A case report

**DOI:** 10.1097/MD.0000000000034519

**Published:** 2023-08-11

**Authors:** Shinichi Takeshima, Toshiki Furuya, Mariko Yamamoto, Marie Noma, Nobuyuki Kawate

**Affiliations:** a Department of Rehabilitation Medicine, Showa University School of Medicine, Kanagawa, Japan; b Department of Internal medicine, Sakuragaoka Central Hospital, Kanagawa, Japan; c Center for Rehabilitation, Showa University Fujigaoka Rehabilitation Hospital, Kanagawa, Japan.

**Keywords:** comprehensive treatment, intensive rehabilitation, neurosarcoidosis, steroids

## Abstract

Neurosarcoidosis tends to prolong the duration of treatment and may result in a decline in physical function requiring rehabilitation. Because of a rare disease, the adjustment of oral steroid dosage, which is the cornerstone of treatment, is highly dependent on professional experience in general. Therefore, the number of hospitals that can perform dosage adjustment is very limited, and it is difficult to provide concurrent intense rehabilitation at the same hospital over a long period of time, and there are no reports that mention this.

**Patient concerns:**

A 49-year-old man, who presented with impaired consciousness, dysphagia and right hemiplegia, was diagnosed with neurosarcoidosis based on a previous diagnosis of sarcoidosis, laboratory test results, and clinical symptoms. High-dose oral steroid therapy was initiated and he was transferred to our rehabilitation hospital for progressive disuse approximately 2 months after the disease onset.

**Diagnoses:**

This case was diagnosed as “probable” neurosarcoidosis.

**Interventions:**

The steroid dose was not reduced during rehabilitation treatment in our hospital considering the risk of relapse of the primary disease due to steroid reduction. His training regimen focused on minimum activities of daily living was performed, and its effectiveness was determined during approximately 60 days after the initiation of rehabilitation.

**Outcomes:**

Two months after admission, he was independently eating, transferring, and toileting under supervision. He was discharged home 3 months after admission.

**Lessons:**

Intensive rehabilitation can be one of the effective comprehensive treatment strategy for patients with neurosarcoidosis. On the other hand, since there is no consensus treatment method, the duration of rehabilitation and goal setting should be adjusted based on an understanding of the characteristics of the disease and the overall treatment plan.

## 1. Introduction

Sarcoidosis is an immune-mediated disease of unknown cause characterized by granulomatous inflammation of various organs, including the lungs in 90% of cases, skin (15%), eyes (10–30%), liver (20–30%), and lymph nodes (10–20%).^[1,2]^ Neurological lesions of sarcoidosis (neurosarcoidosis) may involve the central nervous system, the peripheral nervous system, or both, with a frequency of approximately 5% in patients with sarcoidosis.^[3]^ Sarcoidosis affecting the central nervous system is often treated with potent immunosuppressive therapy. However, because evidence-based treatment guidelines for treatment do not exist, careful handling of each case is necessary. Treatment tends to be prolonged,^[4]^ and patients may require rehabilitation to address the decline in physical function and other disease complications.^[5]^ On the other hand, because of the heavy reliance on the experience of specialists, only a limited number of hospitals are able to adjust the dosage of oral steroids, the cornerstone of treatment. In other words, patients with neurosarcoidosis who require treatment long enough to require rehabilitation must choose between continued dosage adjustment and intense rehabilitation therapy. Unfortunately, few reports mention about this problem.

This case report discusses the potential of rehabilitation therapy as one of the comprehensive treatments for neurosarcoidosis.

## 2. Case presentation

A 49 year-old man developed anterior chest discomfort and respiratory distress at the age of 28 years, and was diagnosed with sarcoidosis via a lymph node biopsy in the hilar region of the lung revealed a noncaseating granuloma. Then he was prescribed a low dose of steroids. Diabetes mellitus was noted after the initiation of treatment for sarcoidosis. Moreover, at 39 years old, he suffered a cerebral infarction, resulting in left hemiplegia; however, he was able to live independently with the use of a short leg brace.

**Figure 1.**
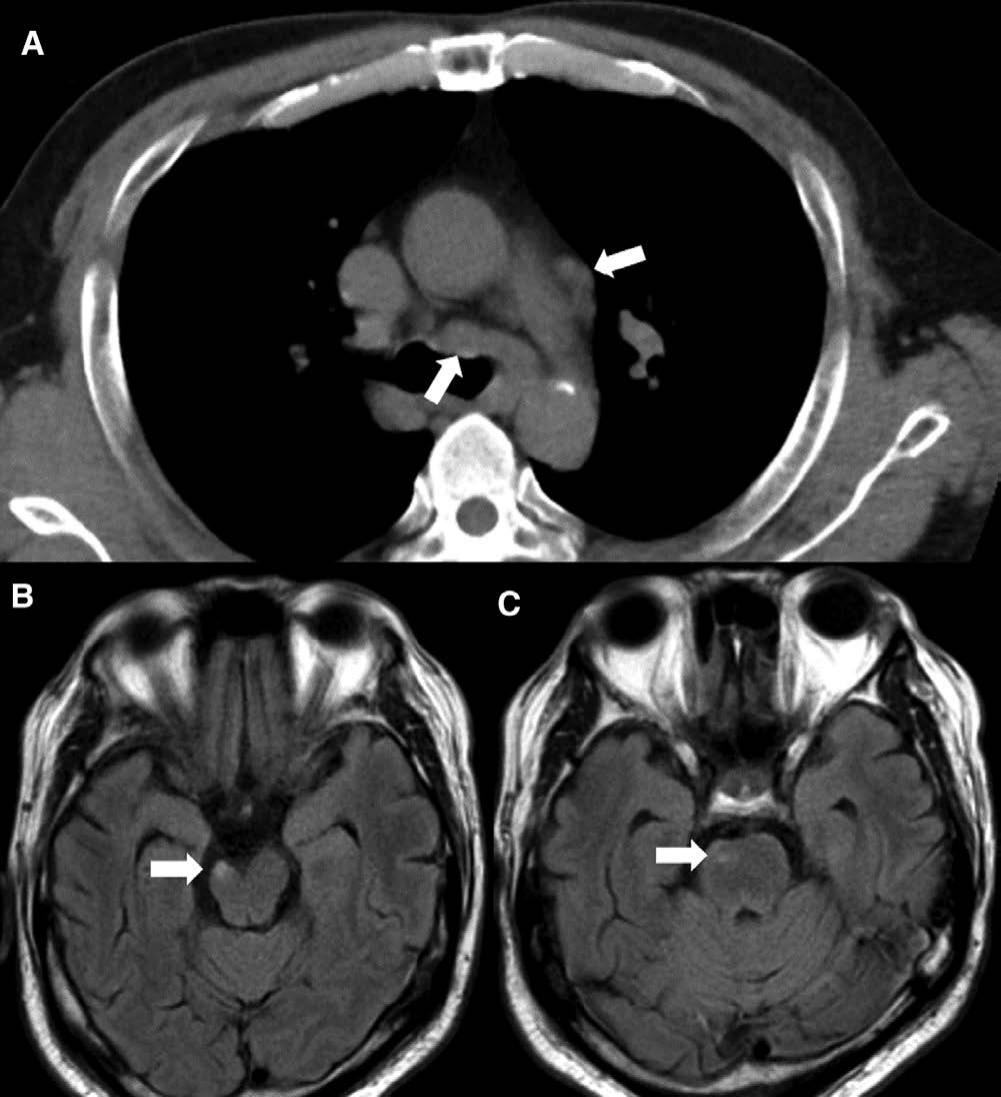
(A) Chest computed tomography. Lymph nodes in the mediastinum are swollen (arrow). (B and C) Head magnetic resonance imaging fluid attenuated inversion recovery images. Although nonspecific as neurosarcoidosis, high-signal areas are founded in the right ventral side of cerebral peduncle (B: arrow) and the pons (C: arrow).

The patient was noted by a family member to have dysarthria on his first sick day for the current illness. The next day, he developed dysphagia and was taken to the emergency hospital. During transport, in addition to his previous left hemiplegia, he exhibited a mild disturbance of consciousness, left abducens nerve palsy, loss of pharyngeal reflexes, and muscle weakness in the right upper and lower limbs. Blood tests were as follows: blood glucose 124 mg/dL, HbA1c 7.9%, albumin 4.2 g/dL, CRP 0.20 mg/dL, leukocytes 9440/µL, Hb 18.2 g/dL, platelets 18.0 × 10^4^/µL, angiotensin converting enzyme 8.1 U/L (normal 7.0–25.0 U/L), IgG 756 mg/dL (normal 820–1740 mg/dL), soluble interleukin-2 receptor 444U/mL (normal 122–496). A lumbar puncture revealed an elevated initial pressure of 290 mm H_2_O (normal 70–120 mm H_2_O). A cerebrospinal fluid (CSF) examination revealed protein 225 mg/dL (normal 10–40 mg/dL), glucose 57 mg/dL (normal 50–75 mg/dL), and cell count 29/μL (normal 0–5/μL), of which 94% were mononuclear cells. Angiotensin converting enzyme in the CSF was 1.2 U/L, and soluble interleukin-2 receptor in the CSF was 386 U/ml. Oligoclonal bands were negative, and the IgG index was 0.51 (normally below 0.73). CSF cultures of bacteria and *Mycobacterium tuberculosis* were negative, as were polymerase chain reaction tests for human herpesvirus and varicella-zoster virus, indicating negative results for meningitis, encephalitis, and other infections. Chest computed tomography revealed a swollen mediastinal lymph node (Fig. 1A).

Head magnetic resonance imaging detected extensive evidence of an old cerebral infarction in the right middle cerebral artery region but no evidence of a new infarction. Fluid-attenuated inversion recovery images revealed a high-signal area in the brainstem (Fig. 1B and C). Neurosarcoidosis was diagnosed due to his history of sarcoidosis diagnosed 21 years prior. Methylprednisone 1000 mg/day was twice administered for 3 days, starting on the 4th and 14th sick days. On the 17th day of illness, prednisone was started at 40 mg/day. After the initiation of treatment, the patient gradually improved in consciousness impairment, abducens nerve palsy, dysarthria, dysphagia, and weakness of the right upper and lower limb muscles. However, he could not perform daily activities and was transferred to our rehabilitation hospital on the 52nd day of his illness for continued intensive rehabilitation treatment.

At the time of admission to the rehabilitation hospital, he had facial paralysis and mild residual dysarthria. Still, his swallowing function and right upper and lower limb muscle strength had generally recovered. Right upper and lower limb muscle strength was generally 3 to 4 on manual muscle testing. His poststroke left hemiplegia was at the Brunnstrom Stage of III–III–IV. The patient had trunk muscle weakness and required full assistance in activities of daily living (ADL), including standing and sitting (Table 1), with a modified Rankin Scale of 5.

**Table 1:** Changes of functional independence measure during hospitalization in the rehabilitation hospital. At about 2 months after admission, improvement was seen especially in transferring, toileting, and dressing.

	53rd sick day	74th sick day	104th sick day
Self-care			
Eating	5	5	6
Grooming	1	3	5
Bathing	1	1	1
Dressing-upper	1	5	6
Dressing-lower	1	2	6
Toileting	1	1	4
Sphincter control			
Bladder	1	4	4
Bowel	1	5	5
Transfers			
Bed, chair, wheelchair	1	3	5
Toilet	1	3	5
Tub, shower	1	1	1
Locomotion			
Walk/wheelchair	1	1	2
Stairs	1	1	1
Communication			
Comprehension	7	7	7
Expression	7	7	7
Social cognition			
Social interaction	7	7	7
Problem solving	7	7	7
Memory	2	7	7

There is no evidence-based treatment for neurosarcoidosis, and the following 3 policies in current patient’s rehabilitation treatment were shared with the patient. First, steroid medication should not be adjusted during intensive rehabilitation considering the risk of relapse of the primary disease due to steroid reduction. Second, to facilitate discharge, the goal is to focus on achieving the minimum necessary activities at home, such as transferring, toileting, and eating. Finally, 1 to 2 months after admission, it should be determined whether a patient can achieve the target physical function. Moreover, if a patient cannot achieve the goals, he should be transferred to a general hospital with a neurology department, where pharmacotherapy can be prioritized.

Since he was taking high-dose steroids, blood glucose control was strictly maintained, masks were worn at all times as part of infection control measures, and training was limited to the same ward. At the beginning of the rehabilitation therapy, training focused on strengthening trunk and lower limb muscles and stabilizing the sitting posture, and encouraged the patient to move away from the bed firstly.

On the other hand, intermittent muscle tonicity was observed at admission in the patient’s right upper and lower limbs. This muscle tonicity was challenging to control voluntarily and interfered with basic movements, such as standing. Since the muscle tonicity extended to the face, he was considered to have epileptic seizures related to neurosarcoidosis, and carbamazepine was started at 200 mg/day on the 60th day of illness. After that episode, his involuntary movements decreased dramatically, and he could perform the daily rehabilitation exercises well. Around the 80th sick day, he stabilized his sitting posture, although he required assistance while standing up. Considering that involuntary movements of the right lower extremity might have affected his standing and walking activities, a short leg orthosis was made for the right lower limb on the 94th sick day. By the 100th sick day (2 months after admission), he could sit independently and transfer and use the toilet under supervision. It was hoped that he could gain physical functions as initially intended (Table 1). After that, achieving those goals and considering his physical function and the family’s ability to help, he limited his living area at home to the area around the bed and introduced a bed handrail and portable toilet. He was discharged home on the 158th sick day, as he could perform minimal ADL. At this time, modified Rankin Scale was 3 and manual muscle testing of the right upper and lower limbs had improved to about 4.After discharge, the patient continued rehabilitation at home and visited the neurologist regularly.

## 3. Discussion

Neurosarcoidosis presents with various symptoms depending on the site of involvement,^[3]^ but the condition does not have definitive laboratory findings. In addition, biopsies of nerve tissue are generally tricky, making the diagnosis very difficult.^[3,6]^ Therefore, it is important to diagnose patients with systemic sarcoidosis that present with neurological symptoms comprehensively, including laboratory findings.^[6]^ Based on the neurological and CSF findings and the previous diagnosis of sarcoidosis, this case was determined as “probable” neurosarcoidosis.^[7]^ The improvement in neurological symptoms after treatment with steroids further supports the diagnosis of neurosarcoidosis.^[7]^ In addition to multiple cranial neuropathies, the current patient had impaired consciousness, right hemiplegia, and convulsive seizures, suggesting that the peripheral nervous system and the central nervous system were affected. Furthermore, based on the variety of cortical symptoms, the lesion was considered a diffuse disseminated intraparenchymal granuloma lesion.

Because neurosarcoidosis is a relatively rare disease,^[3,8]^ case reports are the primary source of information on its clinical spectrum.^[9]^ Furthermore, treatment strategies are limited to expert opinion.^[3]^ Therefore, there have been few reports of rehabilitation treatment as one of the comprehensive treatment measures in parallel with drug therapy, as in the present case.

In light of the above, the following points should be noted when considering the overall treatment plan in this case. First, neurosarcoidosis treatment is an aggressive immunosuppressive therapy centered on steroids, but the clinical symptoms may worsen if steroids are reduced.^[10,11]^ Since the method of dose reduction depends on the experience of the treating physician, steroid dose reduction should have been carefully performed in a specialized department. In previous reports, there were cases in which the steroid dosage was not changed during rehabilitation intervention.^[11,12]^ Steroid dose reduction should be avoided in parallel with rehabilitation therapy because the symptoms of this rare condition cannot be adequately managed in this rehabilitation hospital setting. Second, many cases of diffuse disseminated intraparenchymal granuloma lesions require long-term oral steroid therapy, as in this case.^[8]^ Such long-term, high-capacity steroid therapy is associated with an increased risk of developing steroid-related side effects and severity.^[4,10]^ In this case, because the patient continued to receive prednisone at the starting dose, he had to be adjusted to the lowest effective dose after rehabilitation therapy. Therefore, the purpose of limiting the duration of rehabilitation was to prevent steroid-associated side effects and to facilitate the prompt dose adjustment of the oral medication, the mainstay of treatment, as part of a comprehensive strategy.

This patient responded well to steroids, and symptoms such as impaired consciousness and hemiplegia improved promptly. However, his disuse progressed because of the prolonged treatment period. Therefore, the main focus was on rehabilitation treatment for the deterioration of physical function due to inactivity while managing diabetes, taking infection control measures, and providing countermeasures against epileptic seizures. In particular, in systemic management, the impact of anticonvulsants on the pathophysiology of neurosarcoidosis is still poorly understood.^[4]^ On the other hand, some argue that the pathophysiology of epileptic seizures and neurosarcoidosis do not necessarily coincide,^[13]^ and the appearance of epileptic seizures does not necessarily reflect worsening pathophysiology. Therefore, anticonvulsants should be used aggressively when epileptic seizures inhibit rehabilitation.

A few case reports describe improved physical function following rehabilitation treatment in patients with neurosarcoidosis.^[11,12,14]^ However, all of them were interventions in the acute phase, and the duration of intervention was relatively short. The following factors were cited as contributing to a favorable outcome: early high-dose steroid administration, young age, good health before onset, and early implementation of intensive rehabilitation.^[11]^ This case differs from previous reports in that the patient started intensive rehabilitation more than 50 days after onset, and the treatment strategy differs from previous cases in that the maximum duration of rehabilitation was determined at the start as part of a comprehensive treatment strategy, and drug therapy for symptoms associated with neurosarcoidosis was provided by a single department of rehabilitation medicine. In other words, even if it is not possible to start intensive rehabilitation at an early stage, as long as the underlying disease is controlled by drug treatment, effective rehabilitation can be provided, leading to recovery of physical function. This approach indicates that intensive rehabilitation may be an option for comprehensive treatment for patients with neurosarcoidosis who require long-term treatment.

## 4. Conclusions

Neurosarcoidosis is a rare disease with no consensus treatment plan or long treatment period. Intensive rehabilitation therapy, combined with an accurate plan based on a thorough knowledge of the treatment strategy and clinical manifestations of neurosarcoidosis, may reduce complications from steroids and improve the patient’s ADL and subsequently maintain quality of life.

## Acknowledgments

The authors would like to express our deep appreciation to Kayo Takeshima for her great effort in preparing this report.

## Author contributions

**Conceptualization:** Shinichi Takeshima.

**Data curation:** Shinichi Takeshima, Toshiki Furuya, Mariko Yamamoto, Marie Noma.

**Project administration:** Nobuyuki Kawate.

**Supervision:** Nobuyuki Kawate.
